# Dislocation of Acromio-Clavicular Joint

**Published:** 1912-08-10

**Authors:** A. H. H. Howard


### DISLOCATION OF ACROMIO-CLAVICULAR JOINT.

### Two Cases Treated by Fixation with a Lane's Bone Plate.

By A. H. H. HOWARD, M.R.C.S., L.R.C.P.

*Case I.*—A. P., aged thirty-five, greengrocer, was walking backwards at his horse's head when he fell over, his left shoulder striking the kerb-stone.

When he got up he noticed pain and tenderness on the top of his left shoulder, and he could feel a "lump" there. He was also unable to abduct his arm without pain.

On examination, about one hour later, I found well-marked deformity at the acromio-clavicular joint, due to the raised outer end of the clavicle. It was easily reducible, but returned at once on removal of pressure from above.

Four days later, when bruising, etc., had subsided, the patient was given a general anæsthetic, and I made a horseshoe-shaped incision, with its convexity downwards, over the joint. Muscular and fascial tissues were cut through and retracted till the joint was exposed. The ligaments I found to be ruptured and the outer end of the clavicle displaced upwards.

The torn ligaments were cut off, and the cartilage scraped till fresh bony surfaces were obtained. By the help of an assistant the arm was pushed upwards and the ends of the bones brought into correct apposition. A small hole was bored in the outer end of the clavicle, into which was fixed a screw through one end of a small Lane's bone plate. A similar hole was next bored in the acromion process, and the outer end of the plate screwed to it, thus securing the bones in position. The wound having been washed and dried, the tissues over the joint were brought together in two layers by catgut and the skin incision by a continuous subcuticular silkworm gut suture. The arm was bandaged to the side for four days.

At fourteen days he could abduct the arm to the level of the shoulder, and on the twenty-first day there was no noticeable limitation of movement.

*Case II.*—About one month later, A. W., a middle-aged working farmer, complained to me that six months previously a heavy pole had fallen on his left shoulder. He had, in fact, dislocated the acromio-clavicular joint; it had been treated by bandaging, but the trouble recurred as soon as the bandage was removed. I found abnormal mobility of the outer end of the clavicle, and considerable thickening around the joint. Only about one-third the normal amount of abduction was possible, and this limited mobility greatly interfered with his work. As there was no active inflammation, I operated on him next day, following the same procedure as in the first case. There was chronic inflammatory thickening of the tissues immediately around the site of injury, but less upward displacement of the clavicle than before. He made a good recovery, and, writing on June 22, 1912, the patient says: "I can use my left shoulder freely, but in raising anything heavy above the head it hurts me; or if I move it constantly in a certain direction—viz., in using the hoe—it causes a numb feeling."

In these two cases the fact of fixation of the acromio-clavicular joint does not seem to have caused any great interference with the integrity of the articulation of the shoulder nor marked weakness in arm movement. The use of a bone plate is easier than wiring if operation becomes necessary.

